# Pancreatic stone protein as an early biomarker predicting mortality in a prospective cohort of patients with sepsis requiring ICU management

**DOI:** 10.1186/cc11406

**Published:** 2012-07-02

**Authors:** Yok-Ai Que, Frederik Delodder, Idris Guessous, Rolf Graf, Martha Bain, Thierry Calandra, Lucas Liaudet, Philippe Eggimann

**Affiliations:** 1 Department of Intensive Care Medicine, University Hospital Medical Center (CHUV) and University of Lausanne, Rue du Bugnon 46, CH-1011 Lausanne, Switzerland; 2 Community Prevention Unit, Institute of Social and Preventive Medicine, University Hospital Medical Center (CHUV) and University of Lausanne, Route de la Corniche 10, CH-1010, Switzerland; 3 Unit of Population Epidemiology, Division of Primary Care Medicine, Department of Community Medicine, Primary Care, and Emergency Medicine, Geneva University Hospitals, Geneva, Rue Gabrielle-Perret-Gentil 4, CH-1211, Geneva 14, Switzerland; 4 Swiss Hepato-Pancreatico-Biliary Center, Department of Visceral and Transplant Surgery, University Hospital, Raemistrasse 100, CH-8091 Zürich, Switzerland; 5 Infectious Diseases Service, University Hospital Medical Center (CHUV) and University of Lausanne, Rue du Bugnon 46, CH-1011 Lausanne, Switzerland

## Abstract

**Introduction:**

Biomarkers, such as C-reactive protein [CRP] and procalcitonin [PCT], are insufficiently sensitive or specific to stratify patients with sepsis. We investigate the prognostic value of pancreatic stone protein/regenerating protein (PSP/*reg*) concentration in patients with severe infections.

**Methods:**

PSP/*reg*, CRP, PCT, tumor necrosis factor-alpha (TNF-α), interleukin 1 beta (IL1-β), IL-6 and IL-8 were prospectively measured in cohort of patients ≥ 18 years of age with severe sepsis or septic shock within 24 hours of admission in a medico-surgical intensive care unit (ICU) of a community and referral university hospital, and the ability to predict in-hospital mortality was determined.

**Results:**

We evaluated 107 patients, 33 with severe sepsis and 74 with septic shock, with in-hospital mortality rates of 6% (2/33) and 25% (17/74), respectively. Plasma concentrations of PSP/reg (343.5 vs. 73.5 ng/ml, P < 0.001), PCT (39.3 vs. 12.0 ng/ml, P < 0.001), IL-8 (682 vs. 184 ng/ml, P < 0.001) and IL-6 (1955 vs. 544 pg/ml, P < 0.01) were significantly higher in patients with septic shock than with severe sepsis. Of note, median PSP/*reg *was 13.0 ng/ml (IQR: 4.8) in 20 severely burned patients without infection. The area under the ROC curve for PSP/*reg *(0.65 [95% CI: 0.51 to 0.80]) was higher than for CRP (0.44 [0.29 to 0.60]), PCT 0.46 [0.29 to 0.61]), IL-8 (0.61 [0.43 to 0.77]) or IL-6 (0.59 [0.44 to 0.75]) in predicting in-hospital mortality. In patients with septic shock, PSP/*reg *was the only biomarker associated with in-hospital mortality (P = 0.049). Risk of mortality increased continuously for each ascending quartile of PSP/*reg*.

**Conclusions:**

Measurement of PSP/*reg *concentration within 24 hours of ICU admission may predict in-hospital mortality in patients with septic shock, identifying patients who may benefit most from tailored ICU management.

## Introduction

Despite significant improvements in clinical management, including systematic multimodal approaches [1], severe forms of infections, such as severe sepsis and septic shock, are the leading causes of mortality in patients admitted to the intensive care unit (ICU) [2-4]. Over the last two decades, several promising therapeutic strategies designed to specifically target pathogen or host-related mediators involved in the septic process have failed to reduce mortality [5-7], and this failure is due, in part, to the heterogeneity of both microbial agents and host inflammatory responses [8]. Since therapeutic strategies should be adapted to each individual patient, the identification of patients who are at high risk of death and who might benefit most from early and aggressive treatment would represent a critical step toward such tailored management [9]. Serum biomarkers may assist clinicians in risk stratification and decision-making processes [10]. An ideal biomarker in patients with sepsis should improve early diagnosis and predict early deterioration toward organ failure and eventually death, thereby identifying patients requiring additional aggressive treatments [11]. Owing to a lack of specificity or sensitivity or both (for example, C-reactive protein (CRP) and procalcitonin (PCT) [10,12]) or to a narrow time window of expression (for example, interleukin-6 (IL-6) and IL-8 [8]), currently used biomarkers do not fulfill such requirements. Therefore, further efforts are needed to identify novel sepsis biomarkers.

Pancreatic stone protein/regenerating protein (PSP/*reg*) is constitutively secreted by pancreatic acinar cells into pancreatic juice along with zymogens and is also secreted by subsets of intestinal and gastric cells [13]. Although its precise physiological roles remain only partly defined, it appears to have protective functions by promoting cellular proliferative responses during beta-cell regenerative processes and epithelial repair [13]. PSP/*reg *is upregulated during acute and chronic pancreatitis; in animals, its expression may be induced by stress conditions in the absence of any pancreatic inflammation. In a recent clinical study, serum concentrations of PSP/*reg *were found to be markedly elevated after trauma, and PSP/*reg *concentrations showed a close correlation with the severity of post-trauma infection [14]. Furthermore, PSP/*reg *was found to bind to polymorphonuclear cells and seemed to induce or maintain their activation or both; thus, PSP/*reg *might serve as an acute-phase protein [14].

We therefore hypothesized that PSP/*reg *concentration may represent a potential biomarker of sepsis-related inflammation. To address this hypothesis, we prospectively measured plasma concentrations of PSP/*reg *within 24 hours of ICU admission in 107 patients with clinically diagnosed forms of severe infections. (Some of the results of these studies have been reported in the form of an abstract [15].)

## Materials and methods

### Patient population

This study was performed between February 2008 and June 2010 in a 32-bed adult medico-surgical ICU of a community and referral university hospital. Patients who were at least 18 years old were evaluated within 24 hours of ICU admission for severe sepsis or septic shock. Owing to organizational constraints, inclusion was prospective but could not be strictly consecutive. The study was approved by the Institutional Review Board (Commission cantonale [VD] d'éthique de la recherche sur l'être humain, Lausanne, Switzerland). Written informed consent was obtained from patients or relatives. Infections, sepsis, severe sepsis, and septic shock were defined according to commonly used criteria [16] (Additional file 1). Patients were followed until death or discharge from the hospital. In-hospital mortality was the primary endpoint.

### Data collection

In addition to recording clinical variables collected from the computerized information system (Metavision; IMDsoft, Tel Aviv, Israel), we recorded the age, sex, admission category, origin (home, emergency room, or ward), and McCabe score of each patient [17]. Severity of illness was evaluated on the first day in the ICU by using the Simplified Acute Physiology Score (SAPS) II [18] and III [19] and the Acute Physiology and Chronic Health Evaluation II (APACHE II) score [20]. Organ dysfunction was evaluated by the Sequential Organ Failure Assessment (SOFA) score [21]. Severe comorbidities were described by using definitions of the APACHE II score and SAPS II and III.

### Measurement of plasma biomarker concentrations

At the time of enrollment, heparinized plasma was obtained from each patient for measurements of biomarkers. (Detailed measurement protocols are presented in Additional file 1.) PSP/*reg *was quantified by using an isoform-specific enzyme-linked immunosorbent assay as described previously [14]. Briefly, plasma samples were incubated with plates precoated with guinea pig anti-PSP/*reg *antibody. Rabbit anti-PSP/*reg *was added and subsequently detected by phosphatase-conjugated anti-rabbit immunglobulin G. This assay had a detection limit of less than 0.1 ng/mL and an inter-plate variance of less than 10% [14].

### Statistical analysis

Continuous variables are reported as mean and standard deviation (SD) or medians and interquartile ranges (IQRs) as indicated. Categorical variables are reported as frequencies and percentages. Because the distributions of the biomarkers were skewed, continuous variables between severity of infection or between survivors and non-survivors were compared by using non-parametric two-sided Wilcoxon-Mann-Whitney rank sum tests. APACHE II, SAPS II and III, and SOFA scores are expressed as mean and SD.

Receiver operating characteristic (ROC) curves using concentrations of acute-phase proteins (CRP, PCT, and PSP/*reg*), pro-inflammatory cytokines (IL-6, IL-8, and IL-10), and acute severity scores (APACHE II and SAPS II and III) as independent variables and mortality as a dependent variable were computed first for the entire population and then specifically for patients with septic shock only. From these curves, we computed areas under the curve (AUCs) and 95% confidence intervals (CIs) to assess the discrimination ability of each marker for predictive purposes. Multiple logistic regression analysis and age-adjusted predicted hospital mortality (expressed as age-adjusted beta regression coefficients) were used to assess the relationship (for example, linearity and distribution) between biomarker concentrations and in-hospital mortality. Finally, we estimated the odds ratios (ORs) for mortality and trends across biomarker quartiles. All *P *values were two-sided, and statistical significance was set at a *P *value of less than 0.05. We used Stata (version 11.2; StataCorp LP, College Station, TX, USA) for data processing and analyses.

## Results

### Patient characteristics

From February 2008 to June 2010, 177 patients were prospectively screened (Additional file 2, Figure S1); of these, 25 (14%) declined consent and 18 (10%) died before consent could be obtained. We included 107 patients: 33 (30%) with severe sepsis and 74 (70%) with septic shock (Table 1). There were more males than females in each subgroup. Sixty-one (57%) and 46 (43%) patients presented with community-acquired and nosocomial infections, respectively. Over 75% of patients had pulmonary or abdominal infections, and high percentages were infected with *Streptococcus pneumoniae *(18.7%) or *Escherichia coli *(21.5%). The overall in-hospital mortality rates were 20.5%, 9% for patients with severe sepsis, and 25.7% for patients with septic shock. Most patients had at least one comorbid condition, but fewer than 14% had McCabe scores predicting rapidly fatal outcome.

**Table 1:** Patient characteristics, by sepsis severity (*n *= 107)

Patient characteristic	All	Severe sepsis	Septic shock
	(*n *= 107)	(*n *= 33)	(*n *= 74)
Demographics			
Age in years, mean ± SD	59 ± 17.5	55 ± 20	61 ± 16
Male/Female	62/45	20/13	42/32
Admission categories			
Medical	72 (67%)	24 (73%)	48 (65%)
Scheduled surgery	4 (4%)	0 (0%)	4 (5%)
Unscheduled surgery	31 (29%)	9 (27%)	22 (30%)
Origin			
Community (home/emergency room)	61 (57%)	18 (54.5%)	43 (58.1%)
Nosocomial (hospital transfer)	46 (43%)	15 (45.5%)	31 (41.9%)
Referral from other hospital	20 (18.7%)	5 (15.2%)	15 (20.3%)
Operating room	3 (2.8%)	1 (3%)	2 (2.7%)
Ward	14 (13.1%)	6 (18.2%)	8 (10.8%)
Intermediate care	9 (8.4%)	3 (9.1%)	6 (8.1%)
Severe comorbidities			
Chronic obstructive pulmonary disease	17 (15.8%)	5 (15.1%)	12 (16.2%)
Cardiac insufficiency	16 (15%)	2 (6%)	14 (18.9%)
Cirrhosis	8 (7.5%)	1 (3%)	7 (9.5%)
End-stage renal disease	15 (14%)	3 (9%)	12 (16.2%)
Immunodeficiency^a^	13 (12.1%)	2 (6%)	11 (14.9%)
Insulin-dependent diabetes	6 (5.6%)	1 (3%)	5 (6.7%)
Score, mean ± SD			
APACHE II	30 ± 8	27 ± 86	31 ± 8
SAPS II	71 ± 17	65 ± 16	74 ± 17
SAPS III	76 ± 18	67 ± 21	80 ± 15
SOFA (day 1)	11 ± 3	11 ± 3	11 ± 3
McCabe score			
Non-fatal	63 (59%)	22 (66.6%)	41 (55%)
Ultimately fatal (< 5 years)	30 (28%)	7 (21.2%)	23 (31%)
Rapid fatal (< 6 months)	14 (13%)	4 (12.2%)	10 (14%)
Infection sites			
Pulmonary	37 (34.5%)	14 (42.4%)	23 (31%)
Abdominal	38 (36%)	7 (21.2%)	31 (41.9%)
Bloodstream	7 (6.5%)	4 (12.1%)	3 (4.1%)
CNS and ENT	5 (4.6%)	2 (6.1%)	3 (4.1%)
Urinary tract	5 (4.6%)	2 (6.1%)	3 (4.1%)
Soft tissues	13 (12%)	4 (12.1%)	9 (12.1%)
Miscellaneous	2 (1.8%)	0 (0%)	2 (2.7%)
Microbiology			
Gram-positive (number of positive blood cultures)	41 (38.3%)	17 (51.5%)	24 (32.4%)
*Staphylococcus aureus *(8)	8 (7.5%)	5 (15.1%)	3 (4.1%)
*Streptococcus pyogenes *(2)	10 (9.3%)	2 (6.1%)	8 (10.8%)
*Streptococcus pneumoniae *(8)	20 (18.7%)	8 (24.2%)	12 (16.1%)
Other (2)	3 (2.8%)	2 (6.1%)	1 (1.4%)
Gram-negative	45 (42%)	13 (39.4%)	32 (43.2%)
*Escherichia coli *(14)	23 (21.5%)	5 (15.2%)	18 (24.3%)
Other Enterobacteriacae (5)	7 (6.5%)	1 (3.05%)	6 (8.1%)
*Pseudomonas aeruginosa *(5)	6 (5.6%)	3 (9.05%)	3 (4.1%)
Other (5)	9 (8.4%)	4 (12.1%)	5 (6.7%)
Fungi	3 (2.8%)	0 (0%)	3 (4.1%)
*Candida albicans *(1)	1 (1%)	0 (0%)	1 (1.4%)
Other (0)	2 (1.8%)	0 (0%)	2 (2.7%)
H1N1	1 (1%)	0 (0%)	1 (1.4%)
Undocumented	17 (15.9%)	3 (9.1%)	14 (18.9%)
Outcome			
Hospital mortality	22 (20.6%)	3 (9%)	19 (25.7%)

Values are presented as numbers (percentages) of patients unless indicated otherwise. ^a^HIV, solid organ transplant recipients, hematological malignancies, chronic use of more than 30 mg per day prednisone equivalents. APACHE II, Acute Physiology and Chronic Health Evaluation II; CNS, central nervous system; ENT, ear, nose, and throat; SAPS, Simplified Acute Physiology Score; SD, standard deviation; SOFA, Sequential Organ Failure Assessment.

### Plasma levels of CRP, PCT, PSP/*reg*, and inflammatory cytokines in patients with severe sepsis and septic shock

Plasma concentrations of the various biomarkers in relation to the severity of infection (severe sepsis or septic shock) are presented in Table 2 and in Figure S2 of Additional file 3. In comparison with the concentrations of CRP, those of the acute-phase proteins PCT and PSP/*reg *and the pro-inflammatory cytokines IL-6 and IL-8 were significantly higher in patients with septic shock than in patients with severe sepsis, and the median PSP/*reg *concentration was about five times higher in patients with septic shock (343.5 ng/L) than in patients with severe sepsis (73.5 ng/L). As expected, the acute severity scores (APACHE II and SAPS II and III) were significantly higher in patients with septic shock than in those with severe sepsis.

**Table 2:** Plasma concentrations of biomarkers and severity scores, by sepsis severity

Biomarker or severity scoring system	All (*n *= 107)		Severe sepsis (*n *= 33)		Septic shock (*n *= 74)		
	Median	IQR	Median	IQR	Median	IQR	Test
Acute-phase proteins							
CRP, mg/L	253.5	155.5	218	152	266	158	0.35
PCT, ng/mL	25.12	48.98	11.96	20.94	39.25	49.99	< 0.0001
PSP/*reg*, ng/mL	241.25	399.00	73.5	180.25	343.5	369	< 0.0001
Cytokines							
TNF-α, pg/ml	2.47	7.07	1.93	7.07	2.41	6.97	0.86
IL-1β, pg/mL	6.43	21.04	1.96	15.53	8.175	24.54	0.21
IL-6, pg/mL	1,173.28	6,749.6	544.07	1,237.48	1,755.34	14,514.13	0.004
IL-8, pg/mL	438.95	1,804.67	184.15	408.72	681.775	3,273.42	0.0001
IL-10, pg/mL	149.37	672.82	76.24	239.67	199.94	706.22	0.045
Other							
Leukocytes, G/L	13.5	14.2	13.5	5.5	13.3	17.1	0.28
APACHE II	28	14	26	14	29	14	0.04
SAPS II	69	26	62	29	71	27	0.02
SAPS III	76	27	69	28	79.5	25	0.001
SOFA	11	4	10	5	11	5	0.35

APACHE II, Acute Physiology and Chronic Health Evaluation II; CRP, C-reactive protein; IL, interleukin; IQR, interquartile range; PCT, procalcitonin; PSP/*reg*, pancreatic stone protein/regenerating protein; SAPS, Simplified Acute Physiology Score; SOFA, Sequential Organ Failure Assessment; TNF-α, tumor necrosis factor-alpha.

PSP/*reg *was further measured in the plasma of burned patients who were previously admitted to our ICU and in whom trace element supplementation resulted in improved clinical outcome, including fewer pulmonary infections and better wound healing [22]. In these 20 patients with severe non-infectious inflammation (mean age of 43 ± 16 years, mean total surface body area burned of 45% ± 22%, and median SAPS II of 32 and IQR of 14), the median PSP/*reg *within the first 24 hours of admission was 13.0 ng/mL (IQR of 4.8). Figure S3 of Additional file 4 displays the individual values of PSP/*reg *in these patients compared with those with severe sepsis and septic shock.

### Association between plasma levels of CRP, PCT, PSP/*reg*, and inflammatory cytokines and in-hospital mortality

We assessed the relationship between the plasma concentrations of CRP, PCT, and PSP/*reg*; the concentrations of inflammatory cytokines; the number of leukocytes; severity scores (APACHE II, SAPS II and III, and SOFA); and in-hospital mortality in patients with severe sepsis or septic shock (Table 3 and Additional file 1, Table S1). Overall, regardless of severity, PSP/*reg *concentrations and SAPS III scores were the only variables that differed significantly between survivors and non-survivors (Table 3). Despite their association with severity, IL-6, IL-8, PCT, APACHE II, and SAPS II were not associated with mortality. Among patients with septic shock (Additional file 1, Table S1), PSP/*reg *remained the only biomarker significantly associated with mortality (*P *= 0.049).

**Table 3:** Plasma concentrations of biomarkers and severity scores, by survival status

Biomarker or severity scoring system	All (*n *= 107)				
	Death (*n *= 22)		Survival (*n *= 85)		
	Median	IQR	Median	IQR	*P *value
Acute-phase proteins					
CRP, mg/L	203	188	266	154	0.21
PCT, ng/mL	17.485	51.75	26.22	44.75	0.57
PSP/*reg*, ng/mL	397	435.9	216.1	379	0.02
Cytokines					
TNF-α, pg/ml	2.655	10.36	2.35	6.73	0.46
IL-1β, pg/mL	0.795	34.02	7.6	20.41	0.46
IL-6, pg/mL	2,415.58	16,219.42	790.53	6,467.74	0.16
IL-8, pg/mL	757.775	10,597.73	414.88	1,597.6	0.11
IL-10, pg/mL	416.18	1393.6	136.76	494.89	0.17
Other					
Leukocytes, G/L	13.85	14.45	13.4	14.2	0.27
APACHE II	32	12	27	13	0.4
SAPS II	71.5	26	68	27	0.52
SAPS III	87.5	27	74	25	0.01
SOFA	11	6	11	4	0.9

APACHE II, Acute Physiology and Chronic Health Evaluation II; CRP, C-reactive protein; IL, interleukin; IQR, interquartile range; PCT, procalcitonin; PSP/*reg*, pancreatic stone protein/regenerating protein; SAPS, Simplified Acute Physiology Score; SOFA, Sequential Organ Failure Assessment; TNF-α, tumor necrosis factor-alpha.

### Biomarkers and severity scores as predictors of in-hospital mortality

**Figure 1:**
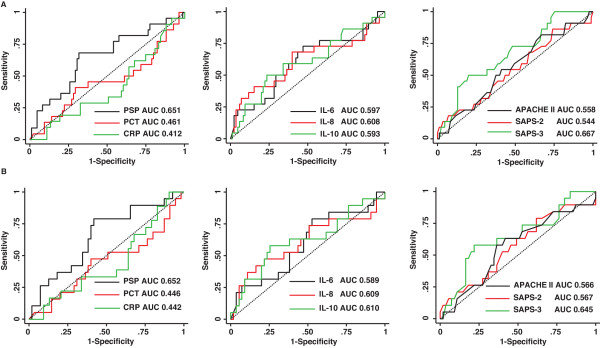
**Receiver operating characteristic (ROC) curves for the entire patient cohort (a) and for patients with septic shock (b)**. Areas under the curve (AUCs) were determined for acute-phase proteins, pro-inflammatory cytokines, and severity scores. APACHE II, Acute Physiology and Chronic Health Evaluation II; CRP, C-reactive protein; IL, interleukin; PCT, procalcitonin; PSP, pancreatic stone protein; SAPS, Simplified Acute Physiology Score; SOFA, Sequential Organ Failure Assessment.

We used AUC analysis to further explore the capacity of each parameter to predict mortality at ICU admission (Figure 1 and Additional file 1, Table S2) within our particular population of patients admitted with a diagnosis of severe sepsis or septic shock. ROCs were computed for the entire patient cohort (Figure 1a) and for patients with septic shock (Figure 1b). When applied to the whole population, PSP/*reg*, IL-8, and IL-6 yielded the highest discriminative value with AUCs (95% CIs) of 0.65 (0.51 to 0.80), 0.61 (0.43 to 0.77), and 0.59 (0.44 to 0.75), respectively. In contrast, the AUCs (95% CIs) of CRP and PCT were 0.44 (0.29 to 0.60) and 0.46 (0.29 to 0.61), respectively (Figure 1a and Additional file 1, Table S2). Similar patterns and AUC values were observed when the analysis was restricted to patients with septic shock (Figure 1b and Additional file 1, Table S2).

**Figure 2:**
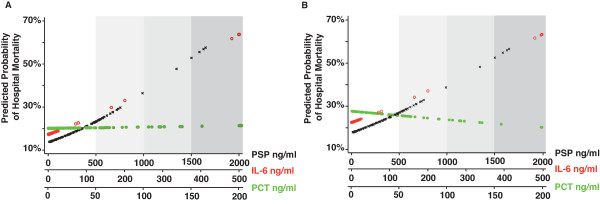
**Probability of in-hospital mortality upon admission among the entire patient cohort (a) and patients with septic shock (b)**. Probabilities are according to their concentrations of plasma pancreatic stone protein/regenerating protein (PSP/*reg*) (crosses), interleukin-6 (IL-6) (open circles), and procalcitonin (PCT) (closed circles). Gradients of grey correspond to quartiles of biomarker concentrations.

PSP/*reg *showed a more uniform and linear distribution than other biomarkers throughout the range of probability of in-hospital mortality (Figure 2a, b). Only seven patients with septic shock (9.5%) presented with IL-6 concentrations in the three highest quartiles in comparison with 20 (27%) for PSP/*reg *(Figure 2b).

The age-adjusted beta regression coefficients - that is, the change in probability of in-hospital mortality per standard unit (one SD) change in biomarker concentration - were 0.16 (SD of 0.07) per 100 ng/mL increase of PSP/*reg *(*P *= 0.03), 0.17 (SD of 0.10) per 50 ng/mL increase of IL-6 (*P *= 0.08), and -0.00076 (SD of 0.0587) per 10 ng/mL increase of PCT (*P *= 0.99). When restricted to patients with septic shock, the age-adjusted beta regression coefficients were 0.16 (SD of 0.08) per 100 ng/mL increase of PSP/*reg *(*P *= 0.048), 0.15 (SD of 0.10) per 50 ng/mL increase of IL-6 (*P *= 0.15), and -0.0167 (SD of 0.062) per 10 ng/mL increase of PCT (*P *= 0.79).

Moreover, in contrast to IL-6 and PCT, the age-adjusted ORs (95% CIs) of mortality among patients with septic shock increased continuously across PSP/*reg *quartiles: PSP/*reg *OR 1.0 [reference group], 1.5 (0.19 to 10.0), 5.1 (0.8 to 32.0), and 6.4 (0.96 to 42.0), respectively (test for trend *P *= 0.02). For IL-6 quartiles, the age-adjusted ORs (95% CIs) for mortality among patients with septic shock were 1.0 [reference group], 2.0 (0.36 to 10.0), 1.3 (0.23 to 7.5), and 2.4 (0.45 to 12.0), respectively (test for trend *P *= 0.41), whereas, for PCT, the corresponding values were 1.0 [reference group], 0.4 (0.7 to 2.0), 0.7 (0.16 to 3.1), and 0.4 (0.08 to 1.9), respectively (test for trend *P *= 0.35).

## Discussion

Our results suggest that PSP/*reg*, a novel acute-phase protein, measured within 24 hours of ICU admission, may predict the risk of mortality in patients with severe sepsis and septic shock. PSP/*reg *was identified in patients with pancreatitis and further associated with islet regeneration [13,23]. The findings that its expression was not restricted to the pancreas [24,25] and that it can be induced by stress in animals [26] opened the way to investigate its role as a potential acute-phase protein. A recent study in 83 trauma patients reported that PSP/*reg *concentrations were 15-fold higher from baseline (5 to 15 ng/mL) in patients developing septic complications [14].

Our results suggest that PSP/*reg*, measured within 24 hours of ICU admission, may be used as a biomarker to identify septic patients at highest risk of death. PSP/*reg *performed better than CRP and PCT, which are widely used to diagnose infection but which, owing to their poor accuracy in this indication, should not be used to predict disease outcome [10,12,27,28]. A similar observation was recently reported in a cohort of 101 patients with ventilator-associated pneumonia [29]. In this cohort, PSP/*reg *measured after 7 days of mechanical ventilation predicted subsequent organ failure development and, eventually, outcome. A major advantage of PSP/*reg *over the currently available predictors of disease evolution, such as cytokines, may be its presence in the plasma of all septic patients at the time of ICU admission. This sharply contrasts with the pro-inflammatory cytokines tumor necrosis factor-alpha, IL-1, and IL-6, which showed very good predictive values in dedicated trials [30,31] but whose use in daily life was finally hampered by their short window of expression in plasma, making them difficult to interpret at the bedside in a population of patients in whom the time between the onset of sepsis and the blood test cannot be standardized [32].

The strength of our study is that it is representative of the usual population of patients admitted to a mixed ICU. Inclusion criteria were simple, making the setting correspond to what any clinician could encounter in his or her daily practice, increasing the chances of PSP/*reg *measurement implementation at the bedside to predict patient outcome. The small number of patients may explain the absence of a correlation of APACHE II or SAPS II with mortality in a cohort of patients with severe infections [33]. In this context, the strong association of PSP/*reg *with mortality might be viewed as indirect evidence of its better specificity for sepsis. The fact that stratification of patients with sepsis could occur within 24 hours of admission would eventually exclude from stratification most patients without a clear diagnosis of sepsis (this strategy resulted in a low number of non-documented infections in our study) as well as those dying rapidly despite resuscitation and for whom very early evaluation of prognosis (< 18 hours) would probably not result in a different initial management [1].

This single-center study also has several limitations. First, since 18 (10%) patients died before consent could be obtained, selection bias cannot be excluded. This may also have contributed to the lower mortality rate of our patients with septic shock compared with rates reported earlier [2-4]. Second, owing to organizational constraints, patient recruitment was restricted to periods when co-investigators worked as attending physicians. Third, since the purpose of the study was to explore the accuracy of PSP/*reg *compared with that of other commonly used or studied predictors (such as pro-inflammatory cytokines) in a homogenous population of patients with a definite diagnosis of sepsis at the time of ICU admission and not between populations of septic and non-septic patients, patients without sepsis were not included. This may have resulted in an apparently modest absolute predicting power (ROC of 0.65). Finally, owing to the relatively small number of patients, these results require further validation in a larger and preferentially multicentric cohort of patients with sepsis.

Though preliminary, our data still suggest that PSP/*reg *determination could be useful in the stratification of patients with septic shock. After initial resuscitation, PSP/*reg *blood levels that are measured within 24 hours of ICU admission may help to detect patients at high risk of death and to reduce the number of those susceptible to receive new and costly adjunctive treatments, such as specific immunotherapy [34], anti-cytokines [35], or extra-corporeal lipopolysaccharide removal [36].

## Conclusions

We conclude that a single measurement of PSP/*reg *concentration within 24 hours of ICU admission stratifies patients with sepsis according to severity of infection and risk of death. If our findings are confirmed by further studies, this promising biomarker may help physicians to tailor treatments to individual patients according to risk of death.

## Key messages

• Commonly used biomarkers, such as C-reactive protein and procalcitonin, are insufficiently sensitive or specific to stratify patients with sepsis.

• Pancreatic stone protein/regenerating protein (PSP/*reg*) is an accurate biomarker to stratify septic patients admitted to intensive care units (ICUs).

• PSP/*reg *blood measurement within 24 hours of ICU admission predicts risk of death more accurately than procalcitonin and pro-inflammatory cytokines.

• PSP/*reg *may help to identify, among all patients admitted for sepsis, those most susceptible to benefit from aggressive management.

## Abbreviations

APACHE II: Acute Physiology and Chronic Health Evaluation II; AUC: area under the curve; CI: confidence interval; CRP: C-reactive protein; ICU: intensive care unit; IL: interleukin; IQR: interquartile range; OR: odds ratio; PCT: procalcitonin; PSP/*reg*: pancreatic stone protein/regenerating protein; ROC: receiver operating characteristic; SAPS: Simplified Acute Physiology Score; SD: standard deviation; SOFA: Sequential Organ Failure Assessment.

## Competing interests

RG is the inventor of a patent that is owned by the University of Zurich and that is for the use of PSP as a 'method for assaying sepsis in humans'. The other authors declare that they have no competing interests.

## Authors' contributions

Y-AQ, IG, TC, LL, and PE were involved in the conception and design of the study, analyzed the data, and wrote the paper. FD collected and analyzed the data. MB and RG measured PSP/*reg *in blood samples and contributed to the analysis and interpretation of data. All authors read and approved the final manuscript.

## Supplementary Material

Additional file 1 ***Supplemental methods*: Definition of sepsis, of infections, of organ dysfunction, of microbiological detection and identification**. Method for measurement of plasma biomarker concentrations [15,37,38]. *Table S1: *Plasma concentrations of biomarkers of sepsis and severity scores in patients with septic shock. *Table S2: *Biomarkers and severity score performances (area under the curve [AUC] values) predicting in-hospital mortality in the entire population of patients with sepsis (ALL) and in the subgroup of patients with septic shock (Septic Shock Patients). Click here for file

Additional file 2 **Figure S1: Study flow chart**. Click here for file

Additional file 3 **Figure S2: Plasma concentrations and medians (dashed lines) of acute phase proteins (Panel A) and pro-inflammatory cytokines (Panel B) within 24 h of ICU admission among 107 patients admitted for severe sepsis (*n *= 33) and septic shock (*n *= 74)**. Click here for file

Additional file 4 **Figure S3: Plasma concentrations of PSP/reg in patients admitted for severe burns, for severe sepsis and septic shock, respectively (Box plot: median, 25th and 75th percentiles, min, max**. Diamond: mean value). Click here for file
