# Supplementary material for: Pancreatic stone protein as an early biomarker predicting mortality in a prospective cohort of patients with sepsis requiring ICU management
Source: Crit Care. 2012 Jul 2;16(4):R114. doi: 10.1186/cc11406 (PMC3580689; doi:10.1186/cc11406)
Supplement: Additional file 1 — Supplemental methods: Definition of sepsis, of infections, of organ dysfunction, of microbiological detection and identification. Method for measurement of plasma biomarker concentrations [15,37,38]. Table S1: Plasma concentrations of biomarkers of sepsis and severity scores in patients with septic shock. Table S2: Biomarkers and severity score performances (area under the curve [AUC] values) predicting in-hospital mortality in the entire population of patients with sepsis (ALL) and in the subgroup of patients with septic shock (Septic Shock Patients). [file cc11406-S1.DOC]

**ADDITIONAL FILE 1**

**SUPPLEMENTAL METHODS**

*Definitions and types of infection.*

Community-acquired infection was defined as infection manifesting before or within 48h after hospital admission, whereas nosocomial infection occurred at least 48h after hospital admission. The site of infection was categorized as pulmonary, abdominal, urinary tract, central nervous system (CNS), ear nose throat (ENT), soft tissue, or bloodstream, with the latter including catheter-related infections, endocarditis and primary bacteremia. Infections were deemed microbiologically documented if a sample collected within a time window ranging from 24 hours before ICU admission to 48 hours after admission yielded a positive culture of pathogenic or potentially pathogenic bacteria [15].

*Microbiological detection and identification*

Identification of each isolate to the species level was performed by various phenotypic tests, such as the catalase test (bioMérieux, Marcy l'Etoile, France), tests with the Slidex Staph plus system (bioMérieux), the oxidase test (Becton Dickinson), the pyrrolidonylarylamidase test (Remel Inc.), and tests with either the Vitek2 or the API system (bioMérieux). Quality control of commercial systems was performed by periodically testing a range of ATCC strains. The criterion used for the acceptance of the identifications obtained with the Vitek2 system was that the isolate was identified as the only choice with a >93% probability. The criteria used for the acceptance of the identifications obtained with the API system were that the isolate was identified with >90% certainty and a typicity index of >0.75. When phenotypic identification failed or was not absolutely certain, sequenced-based molecular identification was performed [36].

*Definitions of sepsis and organ dysfunction.*

Systemic inflammatory response syndrome (SIRS), severe sepsis and septic shock were diagnosed according to the criteria proposed by the 2001 SCCM/ESICM/ACCP/ATS/SIS International Sepsis Definitions Conference . At least two of the following criteria were required for the diagnosis of SIRS: core temperature **>** 38°C or **<** 36°C, heart rate **>** 90 beats/min, respiratory rate **>** 20 breaths/min or PCO_2_ **<** 32 mm Hg or use of mechanical ventilation, and peripheral leukocyte count **>** 12,000 cells /mm3 or **<** 4000 cells /mm3 . SIRS associated with manifestations of infection was categorized as sepsis. Sepsis associated with organ dysfunction or tissue hypoperfusion was considered severe sepsis . Organ dysfunction was defined according to definitions adapted from the SOFA score : cardiovascular system failure was defined as a need for vasoactive and/or inotropic drugs, and/or systolic blood pressure **<** 90 mm Hg, and/or a drop in systolic blood pressure **>** 40 mm Hg from baseline; renal dysfunction was defined as urinary output **<** 0.5 ml/kg/h in patients not previously undergoing hemodialysis for end stage renal failure; respiratory dysfunction was defined as PaO2 **<** 70 mmHg or need for mechanical ventilation or a PaO2/FIO2 ratio of  **<** 250 (or **<** 200 in patients with pneumonia); coagulation abnormalities included thrombocytopenia, defined as a platelet count **<** 80,000/mm3, or INR > 1.5 or aPTT > 60 sec; neurological dysfunction was defined as an altered mental status or a Glasgow coma scale < 15; liver dysfunction was defined as hyperbilirubinemia > 20 mmol/l; the cut-off for elevated plasma lactate was set at **>** 3 mmol/L. Sepsis-induced hypotension was defined as a systolic blood pressure (SBP) < 90 mm Hg or mean arterial pressure < 70 mm Hg or a SBP decrease > 40 mm Hg or < 2 SD below normal for age in the absence of other causes of hypotension. Septic shock was defined as sepsis-induced hypotension persisting despite adequate fluid resuscitation [15, 37].

*Measurement of plasma biomarker concentrations.*

At the time of enrollment, heparinized plasma was obtained from each patient for measurements of biomarkers acute phase protein and pro-inflammatory cytokine concentrations. CRP was measured using the Tina-quant® CRP method on a Modular P apparatus (Roche Diagnostics, Mannheim, Germany) by the Department of Pathology and Laboratory Medicine of the CHUV. PCT was measured using the ELFA method (VIDAS® BRAHMS PCT assay, bioMerieux Inc, Geneva, Switzerland) according to the manufacturer’s instruction, with a detection limit of 0.05 ng/ml and an inter-plate variance of under 20%. Plasma cytokines (TNF-α, IL1-β, IL-6, IL-8, and IL-10) were measured using the Biolegend panel kit and the Luminex method on a Bio-plex apparatus (Biorad, Reinach, Switzerland) at the core facility of the Center for Integrative Genomics, University of Lausanne, Switzerland.

**ADDITIONAL FILE 1: TABLES**

**Additional file 1, Table S1:** Plasma concentrations of biomarkers of sepsis and severity scores in patients with septic shock.

|  | **ALL Septic shock patients** | | | | **Survival** | | | | **Death** | | | |  |
| --- | --- | --- | --- | --- | --- | --- | --- | --- | --- | --- | --- | --- | --- |
|  | **n= 74** | | | | **n=55** | | | | **n=19** | | | |  |
|  | **Median** | | **IQR** | | **Median** | | **IQR** | | **Median** | | **IQR** | | **P** |
| **Acute Phase Proteins** | |  | |  | |  | |  | |  | |  | |
| CRP (mg/L) | 266 | | 158 | | 275 | | 149 | | 220 | | 160 | | 0.46 |
| PCT (ng/ml) | 39.25 | | 49.99 | | 39.31 | | 46.1 | | 39.19 | | 53.72 | | 0.48 |
| PSP/*reg* (ng/ml) | 343.5 | | 369 | | 312.75 | | 400.1 | | 408.25 | | 375.2 | | **0.049** |
| **Cytokines** |  | |  | |  | |  | |  | |  | |  |
| TNF-alpha | 2.41 | | 6.97 | | 2.35 | | 4.98 | | 3.72 | | 18.6 | | 0.41 |
| IL-1β (pg/ml) | 8.175 | | 24.54 | | 8.65 | | 24.23 | | 5.83 | | 60.1 | | 0.84 |
| IL-6 (pg/ml) | 1755.34 | | 14555.41 | | 1559.9 | | 14286.55 | | 3819.89 | | 70194.64 | | 0.25 |
| IL-8 (pg/ml) | 681.775 | | 3273.42 | | 504.93 | | 1777.83 | | 1420.79 | | 14617.46 | | 0.16 |
| IL-10 (pg/ml) | 199.94 | | 658.29 | | 174.95 | | 626.19 | | 583.16 | | 2072.68 | | 0.15 |
| **Other** |  | |  | |  | |  | |  | |  | |  |
| Leucocytes (G/L) | 13.3 | | 17.1 | | 13.2 | | 16.9 | | 13.7 | | 16.7 | | 0.16 |
| APACHE II | 29 | | 14 | | 29 | | 15 | | 33 | | 15 | | 0.36 |
| SAPS II | 71 | | 27 | | 69 | | 28 | | 77 | | 30 | | 0.39 |
| SAPS III | 79.5 | | 25 | | 77 | | 18 | | 94 | | 28 | | 0.06 |
| SOFA | 11 | | 5 | | 11 | | 4 | | 12 | | 8 | | 0.48 |
|  |  | |  | |  | |  | |  | |  | |  |
| IQR: inter-quartile range | |  | |  | |  | |  | |  | |  | |
|  | | | | | | | |  | |  | |  | |

**Additional file 1, Table S2:** Biomarkers and severity score performances (area under the curve [AUC] values) predicting in-hospital mortality in the entire population of patients with sepsis (ALL) and in the subgroup of patients with septic shock (Septic Shock Patients).

|  | **ALL (n=107)** | | | **Septic Shock Patients (n=74)** | | |
| --- | --- | --- | --- | --- | --- | --- |
|  | **AUC** | **95% CI** | | **AUC** | **95% CI** | |
| **Acute Phase Proteins** | |  | |  |  | |
| PSP/*reg* | 0.6508 | 0.51500 | 0.78661 | 0.6517 | 0.50741 | 0.79594 |
| PCT | 0.4610 | 0.31081 | 0.61112 | 0.4459 | 0.28446 | 0.60741 |
| CRP | 0.4119 | 0.27471 | 0.54916 | 0.4423 | 0.28773 | 0.59697 |
| **Cytokines** |  |  |  |  |  |  |
| TNF-alpha | 0.5513 | 0.41133 | 0.69134 | 0.5636 | 0.40003 | 0.72724 |
| IL-1β | 0.4505 | 0.29704 | 0.60403 | 0.4852 | 0.31630 | 0.65403 |
| IL-6 | 0.5968 | 0.45533 | 0.74467 | 0.5885 | 0.43989 | 0.74863 |
| IL-8 | 0.6080 | 0.45132 | 0.76044 | 0.6086 | 0.43739 | 0.77409 |
| IL-10 | 0.5933 | 0.45065 | 0.73598 | 0.6096 | 0.44922 | 0.76922 |
| **Other** |  |  |  |  |  |  |
| APACHE II | 0.5580 | 0.42278 | 0.69326 | 0.5656 | 0.41100 | 0.72010 |
| SAPS II | 0.5439 | 0.40223 | 0.68547 | 0.567 | 0.41126 | 0.72271 |
| SAPS III | 0.6666 | 0.54210 | 0.79105 | 0.645 | 0.49404 | 0.79591 |
| SOFA | 0.5083 | 0.35554 | 0.66103 | 0.5531 | 0.38583 | 0.72039 |

**SUPPLEMENTARY FIGURE LEGENDS**

**ADDITIONAL FILE 2, FIGURE S1:** Study flow chart.

**ADDITIONAL FILE 3, FIGURE S2:** Plasma concentrations and medians (dashed lines) of acute phase proteins (Panel A) and pro-inflammatory cytokines (Panel B) within 24h of ICU admission among 107 patients admitted for severe sepsis (n=33) and septic shock (n=74).

**ADDITIONAL FILE 4, FIGURE S3:** Plasma concentrations of PSP/reg in patients admitted for severe burns, for severe sepsis and septic shock, respectively (Box plot: median, 25^th^ and 75^th^ percentiles, min, max. Diamond: mean value).
