# Supplementary figures and images for: Pancreatic stone protein as an early biomarker predicting mortality in a prospective cohort of patients with sepsis requiring ICU management
Source: Crit Care. 2012 Jul 2;16(4):R114. doi: 10.1186/cc11406 (PMC3580689; doi:10.1186/cc11406)

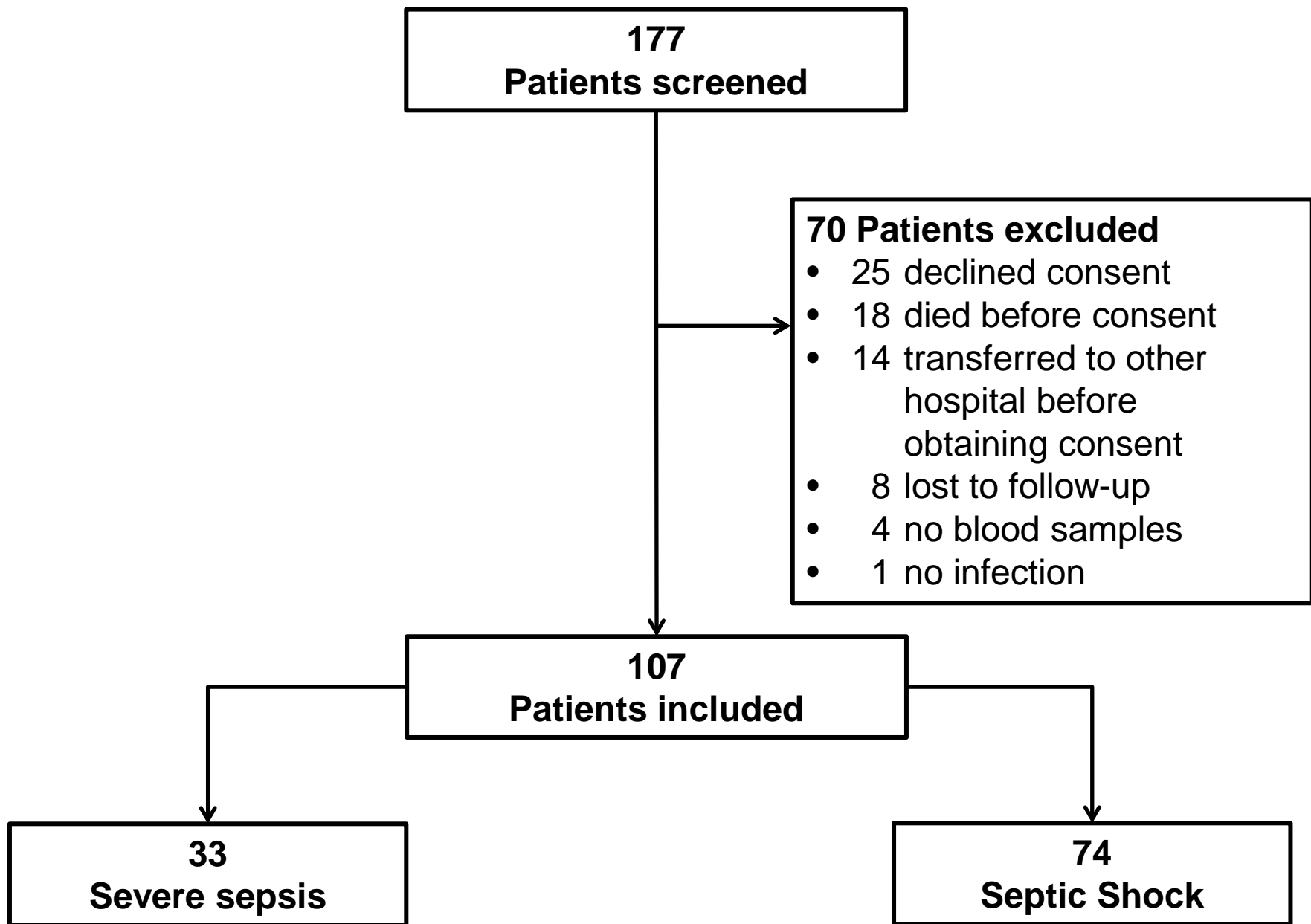

Supplement: Additional file 2 — Figure S1: Study flow chart. [file cc11406-S2.PDF]

A

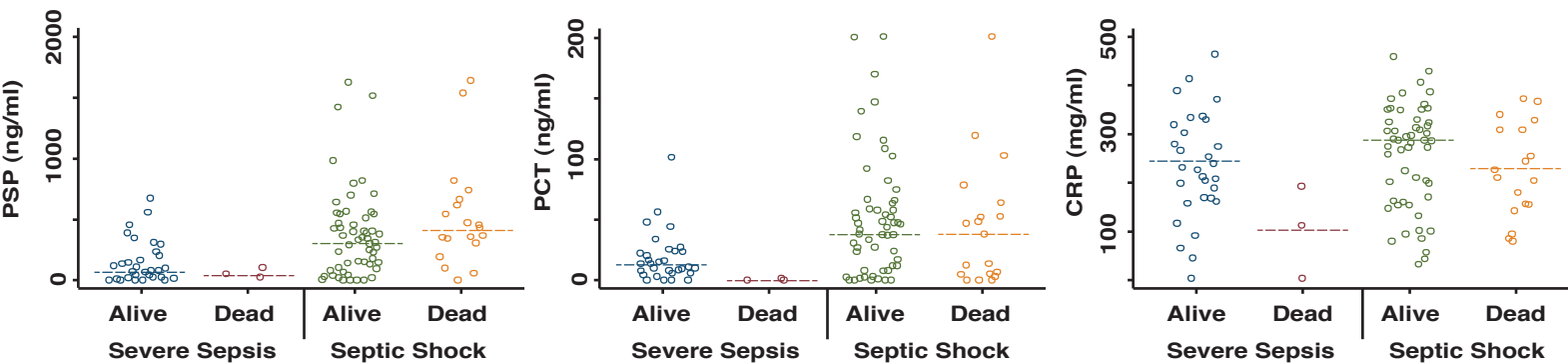

B

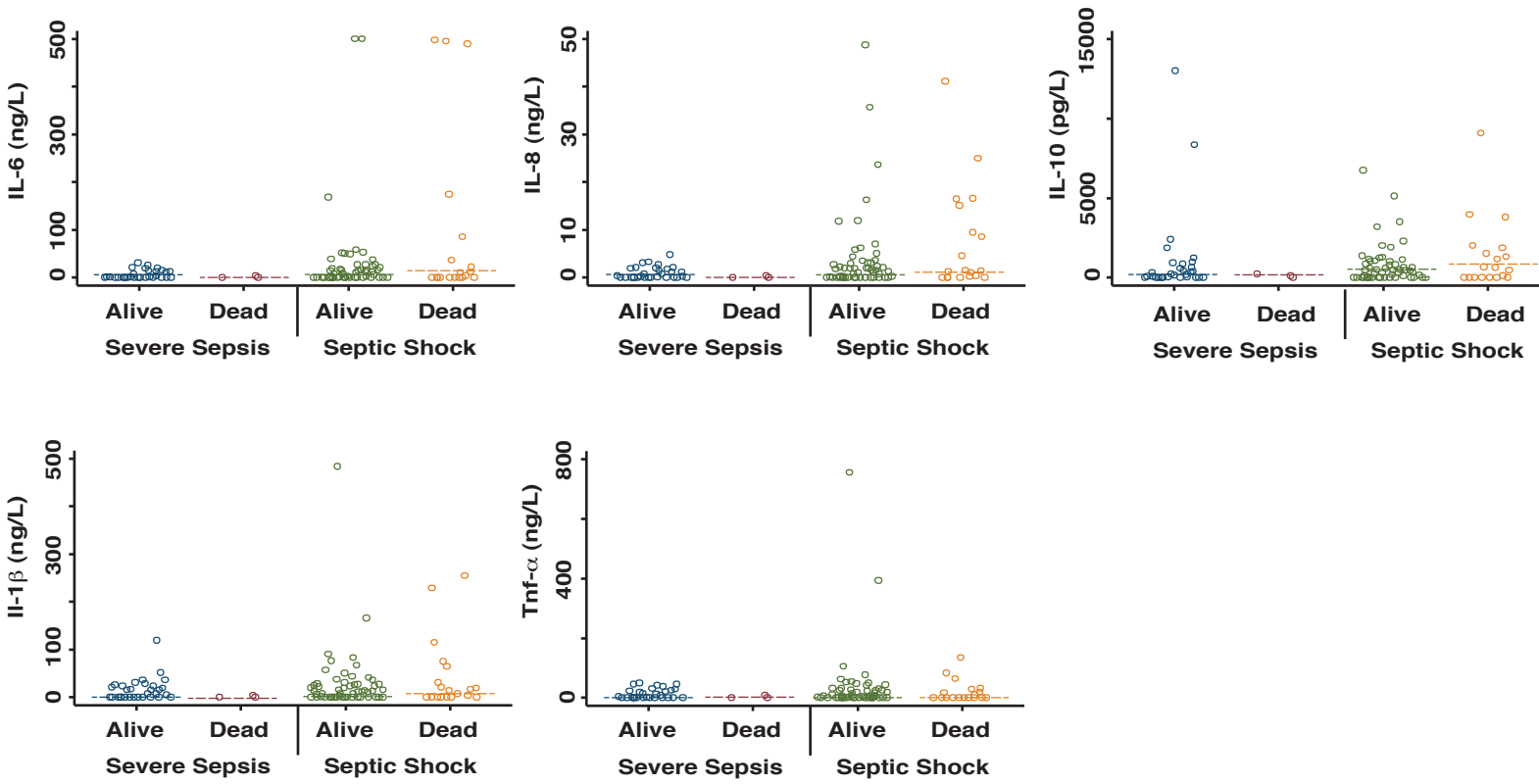

Supplement: Additional file 3 — Figure S2: Plasma concentrations and medians (dashed lines) of acute phase proteins (Panel A) and pro-inflammatory cytokines (Panel B) within 24 h of ICU admission among 107 patients admitted for severe sepsis (n = 33) and septic shock (n = 74). [file cc11406-S3.PDF]

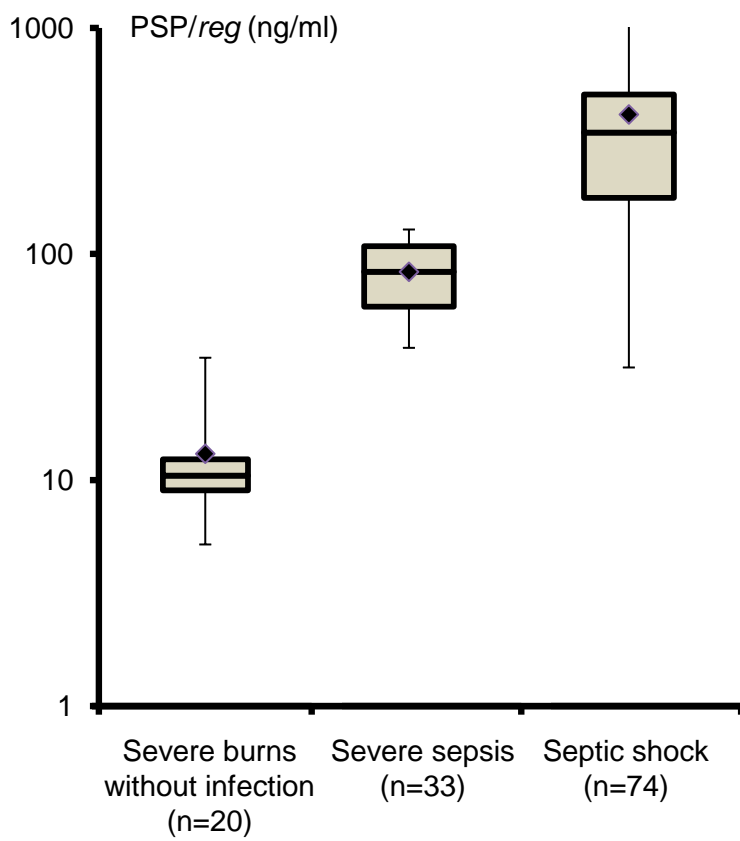

Supplement: Additional file 4 — Figure S3: Plasma concentrations of PSP/reg in patients admitted for severe burns, for severe sepsis and septic shock, respectively (Box plot: median, 25th and 75th percentiles, min, max. Diamond: mean value). [file cc11406-S4.PDF]
